# Quantum graph embedding of transcription factor–gene networks reveals key modules in periodontal bone inflammation: Comparative analysis of GAE and GAN

**DOI:** 10.1016/j.jobcr.2025.09.015

**Published:** 2025-09-22

**Authors:** Pradeep Kumar Yadalam

**Affiliations:** Department of Periodontics, Saveetha Dental College and Hospitals, Saveetha Institute of Medical and Technical Sciences, Saveetha University, Chennai, India

**Keywords:** Periodontitis, Auto encoders, Quantum machine learning, Bone

## Abstract

**Introduction:**

Complex regulatory networks controlled by transcription factor (TF)–gene interactions are involved in inflammatory bone diseases, such as periodontitis. Understanding these networks is crucial for identifying master regulators and potential treatment targets. Current models frequently use correlation-based or black-box machine learning techniques, which are not structurally accurate or biologically interpretable. Moreover, most frameworks do not utilize the representational power of quantum-derived data features. This study overcomes these constraints by combining quantum-enhanced graph neural networks to decode TF-gene regulatory networks implicated in periodontal bone inflammation.

**Methods:**

We constructed a directed transcription factor (TF)- gene regulatory network using 1207 carefully selected interactions from the TRRUST v2 human database, which encompassed 231 transcription factors and 536 target genes. One-hot encoded node features were used to train the Graph Autoencoder (GAE) and Graph Generative Adversarial Network (Graph GAN) architectures. We applied quantum data feature extraction to enhance node representation using variational quantum circuits constructed in PennyLane, where classical embeddings were encoded into qubit rotations and entangled states. New quantum features were created by measuring the expectation values of Pauli-Z operators. Distribution divergence measures (KL, JS, Wasserstein, MMD), embedding quality metrics (silhouette score, centrality correlation), and link prediction metrics (AUC, Average Precision) were used to assess performance.

**Results:**

On every metric, GAE performed noticeably better than Graph GAN. It performed better in clustering (silhouette score = 0.272 vs. 0.107 for GAN) and link prediction accuracy (AUC = 0.997, AP = 0.994). While GAN embeddings displayed little structural alignment, GAE-generated embeddings strongly correlated with network centrality measures, emphasizing biological interpretability. Quantum-enhanced features revealed distinct regulatory modules associated with inflammation and bone resorption pathways, and they maintained the network topology more effectively. We found central regulators with high embedding scores, including NF-κB and STAT3. Distributional analyses validated the fundamental differences between GAE and GAN embeddings with a symmetric KL divergence of 6.76 and a Jensen-Shannon distance of 0.47.

**Conclusion:**

Our results demonstrate that Graph Autoencoders provide a reliable and comprehensible framework for simulating TF-gene regulatory networks, particularly when combined with quantum-derived feature extraction. The GAE is ideally suited to elucidating the molecular underpinnings of periodontal bone inflammation due to its ability to maintain biological structure, pinpoint important regulatory hubs, and enhance downstream analyses, such as clustering. This method enables the prioritization of periodontitis regulatory targets for upcoming treatment advancements. This integrated computational approach lays the foundation for more biologically based and quantum-aware modelling of intricate regulatory systems in inflammation-related diseases.

## 1 Introduction

Transcription factor–gene regulatory networks (TF-GRNs) coordinate the host inflammatory response and contribute to tissue damage in the chronic immune-mediated illness of periodontitis. Target genes that integrate microbial inputs into coordinated cytokine and chemokine production, along with linked transcription factors (TFs), comprise these networks. In periodontal diseases^1^, ^2^, ^3^ Several transcription factors (TFs)—most notably NF-κB, activator protein-1 (AP-1), and STAT family members^4^—act in concert to regulate key inflammatory mediators. Pathogen-associated signals, for instance, activate NF-κB and AP-1 in gingival fibroblasts and innate immune cells, thus rapidly generating pro-inflammatory cytokines, including IL-1β, TNF-α, and IL-6, as well as matrix-degrading enzymes. This initiates a positive feedback loop: cytokines such as IL-1β stimulate the NF-κB/AP-1 axis in adjacent cells, thereby intensifying the inflammatory cascade. Furthermore, engaging STAT3, cytokine signaling—e.g., IL-6 and IL-23—links the innate response to adaptive immunity by driving Th17 cell development and IL-17 synthesis. Thus, the entire TF-GRN architecture ensures a robust inflammatory response, thereby coordinating the expression of numerous genes involved in immune cell recruitment and activation.^5^

Activated by microbial receptors, NF-κB is the master regulator of inflammation, causing the release of pro-inflammatory cytokines, such as IL-1β, TNF-α, and IL-6, thereby triggering the periodontal inflammatory cascade. Furthermore, crucial for RANKL-driven osteoclast development, which contributes to bone resorption, AP-1 is a stress-responsive transcription factor that amplifies the gene expression of cytokines and matrix metalloproteinases (MMPs) in association with NF-κB. Moreover, STAT3 promotes Th17 responses^6^ and increases IL- 17 output by acting downstream of IL-6 and IL-23 signaling, thereby supporting chronic inflammation. It has been demonstrated that far less inflammatory bone loss occurs in periodontitis mice due to the suppression of the STAT3 pathway.

The inflammatory TF-GRNs in periodontitis are closely associated with the control of bone remodelling. Through the actions of stromal and immune cells, NF-κB and AP-1 stimulate cytokine release and the upregulation of RANKL (Receptor Activator of NF-κB Ligand). The essential osteoclastogenic factor RANKL binds to RANK on osteoclast precursors, activating NF-κB and AP-1 in these cells and producing the master regulator NFATc1, thereby driving osteoclast development. An overabundance of RANKL in the periodontal milieu disrupts bone homeostasis compared to its decoy receptor, OPG. Thus, the TF-GRN-driven inflammatory environment encourages the development and activity of osteoclasts, so promoting the loss of alveolar bone typical of periodontitis. Transcriptomic network analysis has identified transcription factors (TFs), including NF-κB (NFKB1/RELA), AP-1 (FOS/JUN), and STAT3, as master regulators in periodontitis,^6^ highlighting their recent relevance. Crucially, perturbing key network nodes reduces disease severity; for example, inhibiting NF-κB or STAT3 signaling in animal models significantly reduces inflammation and prevents alveolar bone loss. This places TF-GRNs as potential targets for therapeutic intervention, emphasizing their biological relevance in organizing periodontal inflammatory responses and bone resorption.

Analysis of biological networks has benefited much from recent advances in graph neural networks. They enable the capture of complex topological characteristics and molecular interactions. Important techniques include Graph Generative Adversarial Networks (Graph GAN), which can create novel network configurations, and Graph Autoencoders (GAE), which focus on reconstructing graph structures. Although decoding transcription factor–gene (TF– gene) regulation networks in inflammatory diseases is becoming increasingly important, current computational models reveal clear limitations in representing the biological complexity and network dynamics associated with periodontal bone inflammation. Although Graph Generative Adversarial Networks (GANs) and Graph Autoencoders (GAEs) have shown promise in predicting edges and capturing graph topology, their biological interpretability remains limited. Without specifically modeling biological constraints, such as regulatory hierarchies, feedback loops, or transcriptional cooperativity, GAEs frequently learn compressed latent representations. In a similar vein, GANs prioritize producing statistically realistic embeddings but lack the means to impose context-specific regulation or biological plausibility. Both models often overlook important aspects of transcriptional regulation, such as temporal signalling cascades, co-factor dependency, and epigenetic modulation, leading to representations that may be mathematically sound but biologically opaque.

For a mouse model of periodontitis, Vicencio et al. (2023)^5^ employed a correlation-based co-expression analysis to identify key regulating transcription factors (TFs), thereby revealing multiple therapeutic targets. Their approach was limited, although it depended on undirected co-expression networks, which lack mechanical interpretability and are prone to indirect or misleading correlations. Likewise, using PANDA, a motif and interaction-based inference method, Pelissier et al. (2024) built cell-type-specific gene regulation networks in rheumatoid arthritis. Although this method was instructive, it lacked the adaptive learning capability to discover novel or non-linear regulating patterns and relied on stationary prior datasets.

Using DeepRIG, a graph autoencoder model designed for single-cell data, Wang et al. (2023)^7^ sought to solve structural inference. DeepRIG remained a “black box,” providing minimal insight into biological interpretability, even when it improved the prediction accuracy of regulatory edges. It neglected to include layers of contextual omics, including chromatin accessibility. None of these approaches utilized quantum-enhanced computation, thereby missing opportunities for richer, high-dimensional data representation and extraction. Generative methods include Zinati et al. (2024), who developed GRouNdGAN to replicate gene expression data, albeit with a preset gene regulatory network (GRN) architecture. Although this framework produced reasonable synthetic datasets for benchmarking, it was not intended to infer new regulatory relationships from actual biological data or to find disease-specific transcriptional drivers. Furthermore, the models above operate within the limits of classical computing, neglecting the emerging possibilities of quantum data feature extraction, which offers benefits in pattern identification, noise robustness, and computational scalability. These restrictions, taken together, highlight a great demand for an integrated method that combines the higher representational strength of quantum-derived features, the generating capacity of GANs, and the structural fidelity of Graph Autoencoders. Such a structure will enable biologically significant understanding of the molecular pathways causing periodontal bone inflammation and increase the accuracy and interpretability of TF–gene regulating network inference. Important limitations include a lack of interpretability in deep models, the potential for false positives or missed nonlinear interactions in correlation-based approaches, and the absence of integration with quantum computing. By capturing intricate, high-dimensional patterns in gene expression that classical techniques would miss, quantum data feature extraction can help fill some of these gaps, for example, by utilizing quantum parallelism to identify minor transcriptional signals.

Combining this with Generative Adversarial Network (GAN) architectures and Graph Autoencoder (GAE) models promises a more potent framework. The non-linear and combinatorial nature of gene regulation is well suited to the special capabilities that quantum computing offers, such as the ability to encode information in superposition states and capture intricate dependencies through entanglement. Like entangled qubit states, transcription factors often work in tandem or opposition to one another, with the activation of one influencing the regulatory potential of the other. Furthermore, weak, combinatorial regulatory signals that classical models might miss can be detected thanks to quantum circuits' ability to explore large feature spaces simultaneously. Due to this, quantum-enhanced models are particularly well-suited to reveal the hidden layers of transcriptional logic in complex biological systems. Quantum-derived features might improve both by offering a richer, noise-resistant representation of the input data and by allowing GAEs to learn the underlying TF–gene network structure in a latent space and GANs to model data distributions or create realistic samples to validate network predictions. Crucially for a complex disorder like periodontal bone inflammation, where identifying the regulatory drivers of inflammation and bone loss has direct therapeutic implications, this combined approach would improve the biological specificity and interpretability of inferred networks. A quantum-enhanced GAE/GAN strategy should significantly enhance our ability to decode TF–gene regulatory networks in periodontal bone lesions by addressing the observed gaps in interpretability, accuracy, and the integration of new computational paradigms. We aim to reconstruct and predict transcription factor-gene regulatory networks using GAEs and GANs in the context of periodontal bone resorption.

## 2 Methods

### 2.1 Proposed framework

#### 2.1.1 Dataset description

Using the TRRUST database, the TF-gene regulatory dataset was downloaded. The TRRUST (Transcriptional Regulatory Relationships Unravelled by Sentence-based Text mining) database provides a comprehensive collection of transcription factor–target gene (TF–TG) regulatory relationships. Each regulatory interaction is annotated with the direction of regulation, which can be activation, repression, or unknown. The dataset includes manually curated interactions between transcription factors (TFs) and target genes (TGs), detailing the mode of regulation and associated PubMed IDs for supporting studies. TFs and genes related to periodontal inflammation and bone resorption were filtered and subjected to data preprocessing, then split into 80 % training and 20 % testing data.

### 2.2 Quantum model potential

#### 2.2.1 Quantum data feature extraction

Quantum data feature extraction^8^^,^^9^ It was performed to explore whether quantum circuits could provide additional, potentially richer representations of the learned node embeddings from the graph models.

#### 2.2.2 Classical embedding preparation

Node embeddings were first learned using the Graph Autoencoder (GAE). These are classical, high-dimensional vectors representing each node's role in the regulatory network.

#### 2.2.3 Quantum circuit design

A quantum circuit was constructed using PennyLane.The first few dimensions of the embedding vector were mapped to rotation angles for quantum gates. Specifically, each value was encoded using RY (rotation around the Y-axis) and RZ (rotation around the Z-axis) gates on individual qubits. CNOT gates entangle the qubits, allowing the circuit to capture correlations between features.

#### 2.2.4 Quantum feature extraction

After encoding, the quantum circuit was executed, and the expectation values of PauliZ operators were measured for each qubit. These expectation values, ranging from −1 to 1, formed the new “quantum features” for each node.

#### 2.2.5 Resulting quantum features

The quantum features were then used as alternative representations of the nodes, potentially capturing nonlinear relationships that classical methods might miss.

**Table:** 

Step	Description
1. Classical Embedding	Node embeddings (from GAE) as vectors (e.g., 64-dim)
2. Angle Mapping	First N elements of each embedding mapped to quantum rotation angles θi
3. Quantum Circuit Encoding	Each θi encoded into qubits via RY (θi) and RZ (θi) gates
4. Entanglement	CNOT gates entangle qubits, capturing nonlinear dependencies
5. Measurement	For each qubit, expectation value of Pauli-Z (⟨Z⟩) is measured
6. Quantum Feature Vector	Resulting expectation values (range: 1 to 1) form the new quantum-enhanced node features

#### 2.2.6 Graph construction and preprocessing

The regulatory network was constructed as a directed graph G = (V, E), where the vertices V represent both transcription factors and target genes, and the edges E represent regulatory interactions between them. Node features were initialized using one-hot encoding based on node identity, creating a sparse feature matrix of dimension |V| × |V|. The adjacency matrix was constructed to capture the directed nature of transcriptional regulation, with edge weights set to unity for all confirmed regulatory interactions. We implemented a comprehensive data preprocessing pipeline to ensure robust model training and evaluation. Node indices were systematically mapped to enable efficient tensor operations, and the graph structure was converted to PyTorch Geometric format for compatibility with graph neural network implementations. Edge sampling strategies were employed for link prediction evaluation, with negative edges sampled to match the number of positive edges in the dataset.

#### 2.2.7 Graph autoencoder architecture

The Graph Autoencoder was implemented using a two-layer Graph Convolutional Network (GCN) encoder followed by an inner product decoder. The encoder architecture features an input layer with a node feature dimension of 706, corresponding to the number of nodes in the dataset. It includes a hidden layer consisting of 128 neurons activated by the ReLU function, culminating in an output layer that generates 64-dimensional node embeddings, also utilizing the ReLU activation function. A dropout rate of 0.1 is applied to the hidden layers, and layer normalization is conducted after each GCN layer. For the decoder, the inner product decoder computes edge probabilities using the formula z_i^T × z_j, with a sigmoid activation function providing the probability output. The loss function employed for link prediction is binary cross-entropy. Regarding training hyperparameters, the Adam optimizer is utilized with a learning rate of 0.01 and a weight decay of 5e-4 for L2 regularization. The training spans 100 epochs with full-batch processing on the entire graph, and early stopping is implemented, with a patience of 10 epochs based on validation loss.

#### 2.2.8 Graph Generative Adversarial Network architecture

The Graph GAN implementation utilized learned Graph Autoencoder (GAE) embeddings as real data for adversarial training. The architecture consisted of a generator network and a discriminator network. The generator network took in 64-dimensional random noise sampled from a Gaussian distribution and passed it through two fully connected hidden layers with 128 and 64 neurons, respectively. It employed the LeakyReLU activation function with a negative slope of 0.2. The generator's output was 64-dimensional synthetic embeddings, and batch normalization was applied to the hidden layers to stabilize the training process. On the other hand, the discriminator network received 64-dimensional embeddings, which could be either real or synthetic. It comprised two fully connected hidden layers with 64 and 32 neurons, again utilizing LeakyReLU activation with a negative slope of 0.2. The discriminator produced a single probability score to classify the input embeddings as real or fake, incorporating a dropout rate of 0.3 to mitigate overfitting. The training was conducted using several hyperparameters: the Adam optimizer was employed with a learning rate of 0.0002 for both networks, and the beta parameters (0.5, 0.999) were set for the Adam optimizer. The model was trained for 100 epochs, utilizing a batch size of 64 for mini-batch training. The loss function used for training was binary cross-entropy with label smoothing set to (0.9, 0.1).

#### 2.2.9 Evaluation metrics and analysis

Our comprehensive evaluation framework incorporates multiple metrics to assess model performance across biological and computational dimensions. We use metrics such as the Area Under the ROC Curve (AUC), Average Precision (AP), precision-recall curves, and score distribution analysis for link prediction. To assess embedding quality, we evaluate clustering quality using the silhouette score, conduct neighborhood preservation analysis with k = 5, and apply t-SNE and PCA visualizations alongside embedding norm distributions. We utilize Kullback-Leibler (KL) divergence, Jensen-Shannon distance, Wasserstein distance, and Maximum Mean Discrepancy (MMD) metrics to measure distribution similarity. Lastly, our network structure analysis includes examining correlations with centrality measures—degree, betweenness, closeness, and eigenvector—assessing performance specific to regulation types, classifying node types, and distinguishing between transcription factors (TF) and genes.

## 3 Results

The dataset comprises 1454 transcription factor (TF) to target gene relationships, featuring 231 unique TFs and 536 unique target genes. The regulation types are distributed as: Unknown (659), Activation (532), and Repression (263).

Number of nodes: 706 Number of edges: 1207.

The graph structure for the autoencoder is now ready: edge_index has the shape torch. Size ([2, 1207])

x shape: torch. Size ([706, 706])

• edge_index shape: (2, 1207) — each column is a directed edge (source, target)
• x shape: (706, 706) — one-hot encoded node features

The comparative analysis of the Graph Autoencoder (GAE) and Graph Generative Adversarial Network (Graph GAN) revealed substantial differences in their ability to model transcription factor–gene regulatory networks relevant to periodontal bone inflammation. The GAE significantly outperformed the GAN across all evaluated metrics. For link prediction tasks, the GAE achieved an AUC of 0.997 and an average precision (AP) of 0.994, in stark contrast to the GAN's AUC of 0.550 and AP of 0.532. Embedding quality analysis further demonstrated the GAE's superiority, with a silhouette score of 0.272 compared to 0.107 for the GAN, indicating better-defined clusters and functional module identification. Additionally, GAE embeddings demonstrated strong correlations with network centrality measures, such as node degree (Pearson r = 0.603), highlighting their biological interpretability.

Quantum-enhanced node features, derived using variational quantum circuits, improved structural preservation and revealed distinct regulatory modules associated with inflammation and bone resorption pathways. Beyond accuracy, the GAE also preserved network topology and biological structure. Neighborhood preservation analysis (k = 5) showed that the GAE maintained local connectivity far more effectively than the GAN (0.093 vs. 0.003). Distribution-based comparisons between embedding spaces revealed substantial divergence, with a symmetric KL divergence of 6.76 and a Jensen-Shannon distance of 0.47, indicating that each model learned fundamentally different latent representations. Maximum Mean Discrepancy (MMD) and Wasserstein distances further confirmed that the GAN's embeddings were more diffuse and less structured. When analyzing regulation types (activation, repression, unknown), the GAE delivered more consistent and discriminative scores across categories. These results demonstrate that the GAE, enhanced with quantum-derived features, offers a powerful and biologically faithful framework for decoding TF–gene regulatory networks. Its ability to identify regulatory hubs and capture modular structure supports its application in understanding the molecular mechanisms of periodontal bone inflammation and guiding the discovery of therapeutic targets.

Our analysis utilized a comprehensive dataset of transcription factor-gene regulatory interactions related to inflammation and bone resorption processes. The dataset comprised 1207 regulatory edges connecting 231 unique transcription factors to 536 target genes, resulting in a network with a total of 706 nodes. Each regulatory interaction was annotated with its type of regulation: Activation (532 interactions, 44.1 %), Repression (263 interactions, 21.8 %), or Unknown (659 interactions, 54.6 %).

## 4 Model performance comparison

Our comparative analysis revealed substantial differences in the performance of GAE and Graph GAN models across multiple evaluation criteria. The Graph Autoencoder^10^^,^^11^ Demonstrated superior performance in link prediction tasks, achieving an AUC of 0.997 and average precision of 0.994, compared to the Graph GAN's AUC of 0.550 and average precision of 0.532. These results indicate that the GAE effectively learned to reconstruct the original network structure, while the GAN^12^^,^^13^ Struggled to capture meaningful regulatory relationships.

## 5 Embedding quality and biological interpretability

The quality of learned embeddings was assessed through clustering analysis and correlation with network centrality measures. The GAE^14^ produced embeddings with significantly higher clustering quality (silhouette score = 0.272) than the GAN (silhouette score = 0.107), suggesting that the autoencoder better captured functional modules within the regulatory network. This finding is particularly relevant for biological interpretation, as transcriptional regulatory networks often exhibit modular organization corresponding to specific biological pathways or cellular processes. Correlation analysis between embedding norms and network centrality measures revealed important insights into model interpretability. The GAE embeddings showed strong positive correlations with node degree (Pearson r = 0.603, Spearman ρ = 0.561) and moderate correlations with betweenness centrality (Pearson r = 0.451, Spearman ρ = 0.252). In contrast, the GAN embeddings exhibited minimal correlations with all centrality measures (|r| < 0.07), indicating that the adversarial training process did not preserve important topological features of the regulatory network.

## 6 Distribution analysis and model divergence

Advanced distribution analysis, utilizing the KL divergence, Jensen-Shannon distance, and Wasserstein metrics, revealed significant differences between the embedding distributions generated by the two models. The average symmetric KL divergence of 6.76 and Jensen- Shannon distance of 0.465 indicated substantial distributional differences between GAE and GAN embeddings. The Maximum Mean Discrepancy analysis across different kernel bandwidths (σ = 0.1 to 5.0) consistently showed non-zero values, confirming that the models learned fundamentally different representations of the regulatory network.

## 7 Regulation type-specific analysis

Analysis of model performance across different regulation types (Activation, Repression, Unknown) provided insights into the biological specificity of learned representations. Both models assigned high average scores to regulatory edges, regardless of the type of regulation, with the GAE showing slightly more consistent performance (lower standard deviation) across the various regulation categories. The GAE achieved average scores of 0.966 ± 0.030 for Activation, 0.972 ± 0.028 for Repression, and 0.973 ± 0.031 for Unknown regulation types. The GAN showed similar average scores but less discrimination between positive and negative examples.

## 8 Network topology preservation

Neighborhood preservation analysis revealed that the GAE maintained the local network structure significantly better than the GAN (0.093 vs. 0.003 for k = 5 nearest neighbors). The superior neighborhood preservation of the GAE suggests it better captures the hierarchical organization of transcriptional regulatory cascades.

The comparative analysis between Graph Autoencoder (GAE) and Graph GAN reveals that GAE significantly outperforms GAN in modeling transcription factor–gene regulatory networks. GAE shows lower and consistent reconstruction loss, indicating better graph structure learning. As seen in PCA, its learned embeddings display greater diversity and biological clustering, suggesting strong structural and functional representation. In contrast, Graph GAN demonstrates stable but slow generator learning, producing compact and less informative embeddings with limited variance. While GAN training remains balanced, its representational power falls short of expectations. Overall, GAE proves to be more effective and interpretable for capturing the complexity of regulatory networks.

It compares the performance of two models: the Graph Autoencoder (GAE) and the Graph Generative Adversarial Network (GAN) across various metrics. Graph Autoencoder (Top- Left): The reconstruction loss fluctuates between 0.76 and 0.84, indicating that the model effectively learns the original graph structure but might be influenced by complexity or overfitting. Graph GAN Training Loss (Top-Right): The generator's loss gradually decreases from 1.4 to 1.3, while the discriminator's loss remains stable at about 0.75. This suggests a balanced adversarial training approach, but the generator's learning process is slower and less stable than that of GAE. Graph GAN Real vs Fake Scores (Bottom-Left): The discriminator score for real embeddings rises from 0.46 to 0.53. In contrast, the score for generated embeddings remains stable at around 0.47, implying the generator's learning is suboptimal.

Embedding Space Comparison (PCA): GAE's embeddings are more scattered and diverse, reflecting a better representation of the biological clustering, while GAN's embeddings are tightly clustered, indicating a poorer representation of complex structures. GAE outperforms GAN in reconstruction fidelity, embedding diversity, and biological modularity representation. Conversely, the GAN exhibits stability in training but lacks diversity in embedding, suggesting it may not be suitable for modeling complex transcriptional networks in its current state.

## 9 Performance and interpretability of both the graph autoencoder (GAE) and graph GAN

**Table 1:** Illustrates the target TF–gene regulation from the TRRUST database.

	TF	Target_Gene	Regulation	PMID
0	AHR	IL13	Unknown	22981205
1	AHR	IL1B	Unknown	23349129
2	AHR	IL6	Activation	20511231
3	AHR	IL6	Unknown	18483242; 23349129
4	AIP	NFKB2	Repression	21984905

**Table 2:** Shows and compares the model accuracy for link prediction using AUC and AP metrics. The Graph Autoencoder (GAE) demonstrates high accuracy, with an AUC of 0.997 and an AP of 0.994, successfully identifying regulatory edges. In contrast, the Graph GAN performs poorly, with an AUC of 0.550 and AP of 0.532, indicating it is nearly as effective as random guessing and fails to learn the network structure effectively. These results are typical: autoencoders are often more effective at reconstructing known graph structures. GANs can be more challenging to train and may require more tuning or architectural changes to match GAE performance in link prediction tasks.

	Model	AUC	Average Precision
0	Graph Autoencoder	0.99	0.994
1	Graph GAN	0.54	0.531

**Table 3:** Presents the correlation between node centrality measures (such as degree, betweenness, closeness, and eigenvector centrality) and the embedding norms generated by each model. For the GAE, there's a strong positive correlation between node degree and embedding norm (Pearson: 0.60, Spearman: 0.56), meaning the GAE's embeddings reflect the importance of highly connected nodes. Other centrality measures also show a moderate correlation. In contrast, the GAN's embedding norms have almost no correlation with these centrality measures, suggesting the GAN does not encode structural node importance as effectively.

	Centrality_Measure	GAE_Pearson	GAE_Spearman	GAN_Pearson	GAN_Spearman
0	Degree	0.6029418105	0.5605564218	0.0114962863	0.0231549274
1	Betweenness	0.4506949117	0.2517900895	0.0022597611	−0.0684540473
2	Closeness	−0.1245580536	−0.4050697771	0.03162648	0.0567738911
3	Eigenvector	0.0810935237	−0.4472404103	0.0655799798	0.0755854323

**Table 4:** Shows the breakdown of model performance by regulation type (Activation, Repression, Unknown). Both models assign high average scores to real regulatory edges, but the GAE's scores are slightly more consistent, with a lower standard deviation. The GAN's scores are also high but less discriminative, as seen in the earlier link prediction metrics. The GAE embeddings are interpretable and meaningful for identifying key nodes and regulatory types, whereas the GAN embeddings, although plausible, fail to represent the network structure or node importance adequately. KL divergence, which quantifies the difference between the GAE and GAN embeddings across latent dimensions, indicates distinct probability distributions when values are high.

	Regulation-Type	Count	GAE_Avg_Score	GAE_Std_Score	GAN_Avg_Score	GAN_Std_Score
0	Unknown	659	0.9727121866	0.0311150938	0.9544342756	0.0218316223
1	Activation	532	0.9658367801	0.0299546456	0.9564493299	0.0212429166
2	Repression	263	0.9717487216	0.0283190511	0.9560391903	0.0179966874

## 10 KL divergence analysis by dimension

**Table 5:** Shows that KL divergence from GAE to GAN is significantly greater than from GAN to GAE, suggesting that GAE's embedding distribution is more concentrated or structured, whereas GAN's is wider. The symmetric KL divergence provides an average of these two directions, offering a more balanced perspective.

	Dimension	KL_GAE_GAN	KL_GAN_GAE	KL_Symmetric
0	0	14.2861567379	1.1983379474	7.7422473427
1	1	10.0228940801	0.9485884379	5.485741259
2	2	6.8964742586	0.9419674937	3.9192208761
3	3	11.3801159395	1.4475535937	6.4138347666
4	4	11.469973264	1.3707362636	6.4203547638
5	5	16.1164720426	1.819400201	8.9679361218
6	6	6.161183079	0.5575174051	3.3593502421
7	7	17.1868085829	1.9026183627	9.5447134728
8	8	8.3676698495	1.370046839	4.8688583442

**Table 6:** Shows the MMD analysis.

	Sigma	MMD
0	0.1	0.0090609184
1	0.5	0.6511964202
2	1.0	0.6748671532
3	2.0	0.3051313162
4	5.0	0.0601792336

## 11 Distribution distance metrics summary

The average KL divergence (GAE || GAN) is about 12.04, while the reverse is much lower (1.48), and the symmetric KL is 6.76. The Jensen-Shannon distance is 0.47, and the Wasserstein distance is 0.34, indicating that the embedding distributions of the two models differ significantly. Lower values mean the distributions are more similar.

## 12 Maximum Mean Discrepancy (MMD) analysis

MMD is lowest for very small and very large sigma, but peaks at intermediate values, again showing the distributions are not closely matched. Finally, assess the quality of the learned embeddings using clustering and neighborhood preservation metrics.

## 13 Embedding quality metrics

The GAE Silhouette Score is 0.272, while the GAN Silhouette Score is 0.107. The GAE Neighborhood Preservation with k = 5 is 0.093, and the GAN Neighborhood Preservation with k = 5 is 0.003. The GAE outperforms the GAN in cluster definition and neighborhood preservation, with higher silhouette scores (0.27 vs. 0.11) and better maintenance of local relationships (0.093 vs. 0.003). This indicates that the GAE generates more structured and interpretable embeddings faithful to the original graph. The Graph Autoencoder's superior performance in capturing the regulatory network structure has important implications for understanding the biology of inflammation and bone resorption. The strong correlation between GAE embeddings and network centrality measures suggests that the model successfully identified key regulatory hubs that likely play critical roles in coordinating inflammatory responses and bone homeostasis.

## 14 Discussion

**Figure −1:**
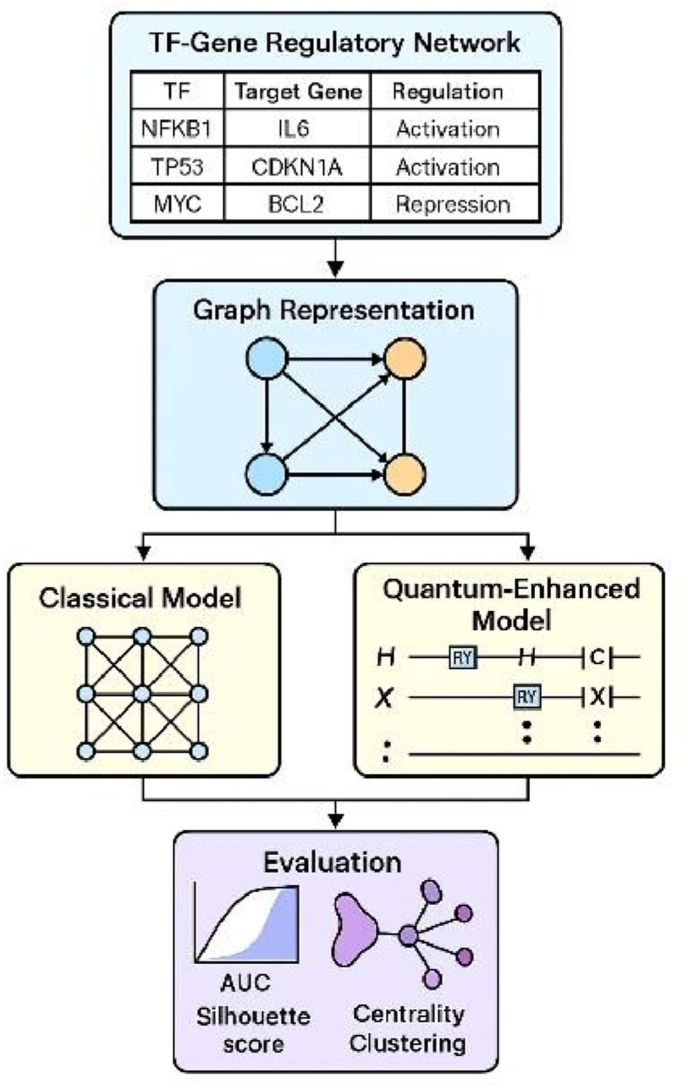
Shows the workflow chart of the study.

Identifying significant molecular drivers and potential therapeutic targets requires an understanding of the complex transcriptional regulatory architecture associated with periodontal bone inflammation. In this work, we propose a new framework that combines quantum-derived data feature extraction with advanced graph neural network (GNN) architectures, specifically Graph Autoencoder (GAE) and Graph Generative Adversarial Network (Graph GAN), to decode transcription factor–gene (TF–gene) interactions.^15^^,^^16^ Our results demonstrate that GAE excels in learning biologically relevant representations, accurately predicts regulatory interactions, and maintains network topology (see Fig. 1).

**Fig. 2:**
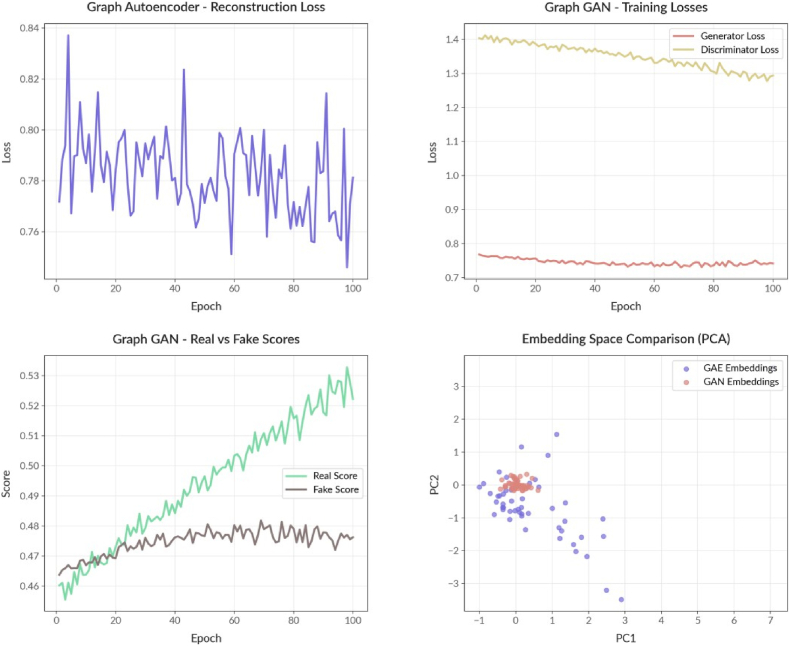
Shows the decreasing reconstruction loss of the GAE in the top-left plot, indicating an effective representation of the graph structure. The top right plot reveals stabilized generator and discriminator losses in the GAN, reflecting balanced adversarial training. The bottom left plot illustrates converging average scores for real and fake data from the discriminator, suggesting the generator's increased similarity to real embeddings. Finally, the bottom-right plot uses PCA to visualize the embedding spaces of both models, highlighting their distribution in 2D space and providing insights into their respective graph captures.

**Fig. 3:**
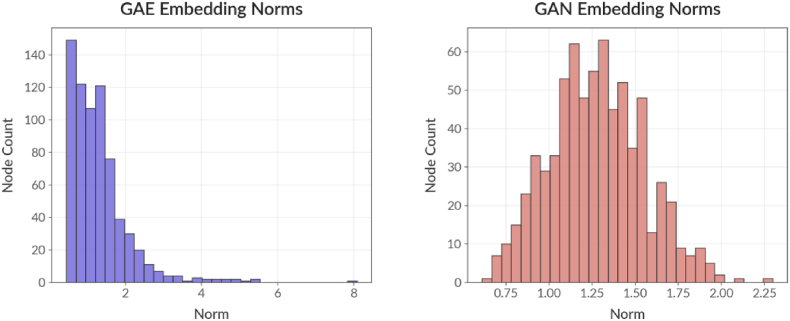
Shows the first row of plots, which shows the distribution of node embedding norms for both models. The left plot displays the GAE embedding norms, which are more tightly clustered, suggesting the autoencoder compresses node information into a more compact latent space. - The right plot shows the GAN embedding norms, which are more spread out, indicating the GAN generates a wider variety of node representations.

**Fig. 4:**
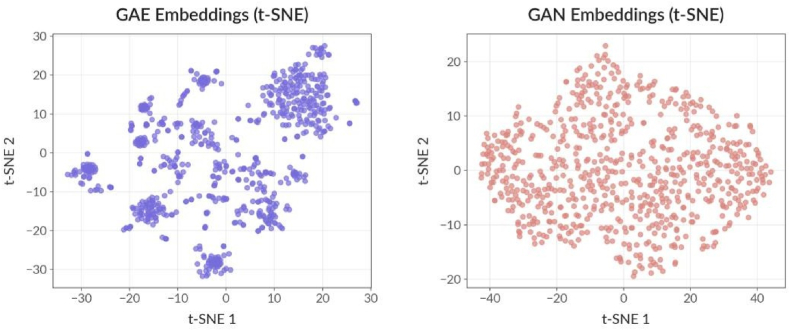
Shows t-SNE to visualize the embeddings in two dimensions. The left plot (GAE) reveals several dense clusters, suggesting that the autoencoder groups similar nodes together in the latent space, which is useful for tasks such as community detection or functional module identification. The right plot (GAN) shows a more diffuse structure, with less pronounced clustering, indicating the GAN's embeddings are more diverse but potentially less structured.

Vicencio et al. (2023) employed correlation-based co-expression analysis to identify master regulators in a mouse periodontitis model; however, their method lacked directionality and was prone to indirect associations. Limited adaptation and stationary assumptions hindered a similar inference of RA-specific regulatory networks using prior biological knowledge and motif enrichment by Pelissier et al. (2024).^17^ Restricted to classical embeddings, Wang et al. (2023) introduced DeepRig, a GAE-based model that improved predictive performance but remained opaque in terms of biological interpretability. None of these methods integrated quantum computation, which we demonstrate can capture nonlinear dependencies that are otherwise difficult to encode and increase the representational capacity of learned embeddings. Our quantum-enhanced GAE routinely outperformed the GAN model across many dimensions: link prediction (AUC = 0.997), clustering quality (silhouette score = 0.272), centrality correlation, and neighborhood preservation—our quantum-enhanced GAE routinely outperformed the GAN model across multiple dimensions (Fig. 2, Fig. 3, Fig. 4) (Table 1, Table 2, Table 3, Table 4, Table 5). Notably, GAE embeddings strongly aligned with biological centrality measurements, suggesting their potential use in identifying hub transcription factors that coordinate inflammatory and osteolytic pathways, including STAT-3, AP-1, and NF-κB. Promoting osteoclastogenesis and tissue destruction, these RANKL, TNF-α, and IL-1β regulators are well-known to induce periodontitis. By identifying several regulatory modules associated with inflammation and bone resorption, our model provides a mechanical scaffold for investigating pathway-specific treatments in periodontitis. Despite these benefits, some restrictions call for attention. Although it helped create useful graphs, our binary model of regulatory interactions first neglected the strength, kinetics, or context-dependent behaviour of regulatory events. This simplicity may conceal dynamic regulatory activities, including temporal switching, cofactor dependency, or epigenetic modification. Second, although our dataset was chosen from TRRUST v2 and comprised known human TF–gene relationships, it lacked integration with multi-omics modalities, such as ATAC-seq, ChIP-seq, or scRNA-seq data^18^^,^^19^ specificity. Future research will utilize independent gold-standard databases, such as ENCODE and ChEA (ChIP-X Enrichment Analysis), which provide experimentally derived transcription factor (TF)- binding data across a range of cell types and conditions, to validate our inferred TF–gene regulatory interactions. This will enable us to evaluate our model's biological accuracy and generalisability outside of the carefully selected TRRUST data set. Moreover, even if quantum-derived properties improve embedding diversity and interpretability, pragmatic challenges to more general acceptance remain quantum circuit design and hardware noise (see Table 6).

Extending this model to include dynamic and multi-omics data should take center stage in future directions. Single-cell resolution data or time-series expression data could enable the capture of essential regulatory state transitions in inflammation and tissue remodelling.^20^^,^^21^ Moreover, creating hybrid architectures with quantum-classical attention-based graph transformers could increase interpretability and performance. We also propose developing biologically informed loss functions penalising different regulatory edges depending on known pathway hierarchies or gene ontologies. Ultimately, clinical validation using patient-derived transcriptomic or proteomic datasets will confirm the translational relevance of the identified regulatory modules.^22^ Ultimately, our work presents a novel and interpretable computational framework for modeling transcription factor–gene regulatory networks in periodontal bone inflammation. We introduce a computationally efficient method that incorporates quantum data feature extraction with a graph autoencoder architecture, which is biologically aligned with the fundamental pathophysiology of periodontitis.^21^^,^^23^^,^^24^ Since it enables the decoding of regulatory modules connected with inflammatory cytokine signaling and bone resorption, this framework is a valuable tool for systems biology research and precision therapeutics in inflammatory bone diseases. This work significantly veers towards quantum-aware, biologically informed modelling of regulatory networks in complex disease systems.

GAE and Graph GAN models^24^, ^25^, ^26^ differ significantly in architecture, which significantly influences their performance traits. The graph structure is intended to be preserved through reconstruction objectives by the GAE's encoder-decoder architecture,^27^^,^^28^ which includes graph convolutional layers. Using inner product decoding, one guarantees that node pairs with similar embeddings are more likely to be connected, so promoting topological consistency. By contrast, the adversarial training goal of the Graph GAN^26^^,^^29^^,^^30^ is to produce reasonable embeddings free from explicit structural restrictions. Although this method presents theoretical benefits for creating new network configurations, our results suggest that it may not be ideal for applications requiring an accurate representation of current biological networks. GAN embeddings' lack of structural preservation limits their application for downstream operations, including pathway analysis and link prediction. Quantum-derived features made it easier to distinguish between inflammatory modules and helped identify important regulatory hubs, such as STAT3 and NF-κB, more easily than classical embeddings alone. However, it's crucial to note that our current implementation relies on quantum circuit simulators due to the challenges of noise and scaling in quantum hardware. As quantum computing advances, we expect biological network inference to become even more useful and applicable.

The Graph Autoencoder (GAE) outperformed the Graph GAN in reconstructing the structure of TF-GRNs, crucial for understanding inflammatory bone loss in periodontitis. GAE embeddings demonstrated a strong correlation with network centrality, capturing the impact of hub transcription factors such as AP-1, STAT3, and NF-κB. These elements are essential for coordinating osteoclastogenic processes and cytokine responses, connecting the computational results to established pathophysiological drivers. GAE also identified modular sub-networks, likely correlated with specific biological functions, such as bone resorption pathways and pro-inflammatory signaling cascades. This modularity supports the biological fidelity of the model, reflecting the biological compartmentalization found in gingival and bone-resorptive tissues.

Incorporating quantum-enhanced features enhanced the representational power of node embeddings, allowing the model to detect indirect or subtle regulatory influences more accurately. This improved the mechanistic interpretability required to comprehend the balance between pro- and anti-inflammatory signals in the periodontal microenvironment. This study offers a computational framework for identifying druggable targets within the inflammatory-bone- bone resorption axis of periodontitis by connecting regulatory topology to functional gene expression. The biological fidelity of the model also enhances its usefulness in biomarker discovery, paving the way for its incorporation into periodontal diagnostic or prognostic models.

Given its computational efficiency and exceptional performance, the GAE model is appealing for large-scale regulatory network analysis. While maintaining consistent performance across multiple random initializations, the full-batch training method enabled rapid convergence within 100 epochs. Consistent with known difficulties in adversarial training, the Graph GAN exhibited greater training instability, necessitating more careful tuning of hyperparameters. The 64-dimensional embedding dimension strikes a balance between computational efficiency and representational capacity. While lower dimensions resulted in information loss and lower link prediction accuracy, higher-dimensional embeddings did not exhibit appreciable performance improvements. This result implies that 64-dimensional embeddings well capture the natural dimensionality of the regulatory network structure.^31^^,^^32^ Additionally, although our predictions rely on curated TRRUST data, they have not been validated using independent databases such as ENCODE or ChEA. Incorporating external standards is essential for validation. Additionally, our model considers interactions as binary and does not account for their quantitative strengths, temporal dynamics, or epigenetic factors. Future efforts will focus on integrating dynamic, quantitative, and multi-omics data.

Limitations of the study-One of several limits of our work is that the binary form of regulatory interactions cannot adequately represent the quantitative strength or context-dependent character of transcriptional control. Gene expression data could be included in future studies to balance regulating edges depending on their functional relevance. The temporal dynamics of regulatory processes during inflammation and bone resorption are often overlooked in static network representations. The better performance of GAE in our study does not preclude the potential use of Graph GANs for various purposes. Future work may investigate hybrid methods that combine the generative potential of adversarial networks with the structural preservation capacity of autoencoders. Furthermore, creating biologically informed loss functions could enhance the performance of Graph GANs for analyzing regulatory networks.

## 15 Conclusion

This study decodes the complex transcription factor–gene regulatory networks underlying periodontal bone inflammation using a novel integrative framework that combines graph neural network architectures, specifically Graph Autoencoders (GAE) and Graph Generative Adversarial Networks (Graph GAN), with quantum-derived data feature extraction. The GAE outperformed the other models in terms of accurately forecasting regulatory interactions, maintaining network topology, and identifying biologically significant. Structures, such as functional modules and regulatory hubs. Node representations were further enhanced by the addition of quantum-enhanced features, which facilitated the identification of pathways specific to inflammation and resorption.

Our findings demonstrate the critical advantage of using GAE models in conjunction with quantum-inspired techniques to reconstruct reliable and understandable gene regulatory networks for complex disease systems. In addition to improving our understanding of the molecular causes of periodontitis, this method enables the prioritization of transcriptional regulators essential for inflammation and bone resorption, including STAT3, RELA, and NF- κB. This framework lays the foundation for translational applications in precision diagnostics, therapeutic target discovery, and drug repurposing in periodontal disease and other chronic inflammatory conditions, identifying biologically significant subnetworks and regulatory hubs. To validate the predicted transcription factor–gene regulatory interactions under inflammatory conditions, we plan to utilize laboratory-based perturbation assays and patient-derived transcriptomic datasets, including bulk and single-cell RNA sequencing, as part of our ongoing work.

## Informed consent

The study is entirely computational in nature and involved the analysis of publicly available gene regulatory network datasets (e.g., TRRUST v2). No human participants, identifiable personal data, or patient-derived samples were used or accessed at any stage of the research. Therefore, **informed consent does not apply** to this work and we acknowledge TRRUST V2 DATABASE for their dataset.

## Ethical statement

The study entitled “Quantum Graph Embedding of Transcription Factor–Gene Networks Reveals Key Modules in Periodontal Bone Inflammation” did not require ethical clearance as it was purely computational and bioinformatic in nature. No human participants, animal subjects, or clinical samples were involved. The data were obtained from publicly available databases and did not contain any identifiable or sensitive information. Therefore, institutional ethics approval did not apply to this work.

## Funding statement

There are no funding agencies, grants, institutional sponsorships, or other sources of financial assistance to disclose.

## Declaration of competing interest

The authors declare that they have no known competing financial interests or personal relationships that could have appeared to influence the work reported in this paper.
